# Fibroblasts Exposed to Serum Collected After Long-Term Calorie Restriction Have Lower Mitochondrial Respiration

**DOI:** 10.1093/geroni/igaf122.2543

**Published:** 2025-12-31

**Authors:** Lek Wei Seow, Stephen Dozier, Nina Sun, Jaclyn Bergstrom, Nathan LeBrasseur, Anthony Molina

**Affiliations:** University of California San Diego School of Medicine, Division of Geriatrics, Gerontology, and Palliative Care, La Jolla, California, United States; University of California San Diego, San Diego, California, United States; University of California San Diego, San Diego, California, United States; University of California San Diego, San Diego, California, United States; Mayo Clinic, Rochester, Minnesota, United States; University of California San Diego, La Jolla, California, United States

## Abstract

Multiple lines of evidence suggest that calorie restriction may slow biological processes related to aging and extend lifespan. To determine whether calorie restriction affects mitochondrial function, we treated fibroblasts from adult donors with serum samples obtained from participants of the CALERIE (Comprehensive Assessment of Long-term Effects of Reducing Intake of Energy) study. The CALERIE study enrolled healthy, non-obese middle-aged (average 38y.o) participants to examine the effects of long-term calorie restriction. We studied the serum samples of 177 participants (calorie restriction CR = 121, ad libitum AL = 56) who had specimens available at two timepoints (12 and 24 months). We assessed the basal and maximal mitochondrial respiration of fibroblasts treated with dilute serum for 24 hours. Our results show that the baseline basal and maximal mitochondrial respiration of fibroblasts is positively correlated with the adiposity of the serum donor (R = 0.112, P = 0.137; R = 0.183, P = 0.015, respectively). Interestingly, treatment of fibroblasts with serum collected after 12 months of calorie restriction causes a decrease in both basal and maximal mitochondrial respiration (P = 0.1854; P = 0.0623, respectively). Fibroblasts treated with serum collected after 24 months of calorie restriction exhibited a further reduction in both basal and maximal mitochondrial respiration (P = 0.0174; P = 0.0092, respectively). These findings suggest that the serum of individuals on long term calorie restriction diet comprises factors that can reduce oxidative phosphorylation. This reduction in bioenergetic capacity may align with the theory that calorie restriction leads to a cellular energy-conserving state, which could have implications for cellular aging and metabolism.

